# Novel role of extracellular matrix protein 1 (ECM1) in cardiac aging and myocardial infarction

**DOI:** 10.1371/journal.pone.0212230

**Published:** 2019-02-21

**Authors:** Sean A. Hardy, Nishani S. Mabotuwana, Lucy A. Murtha, Brianna Coulter, Sonia Sanchez-Bezanilla, Mohammed S. Al-Omary, Tharindu Senanayake, Svenja Loering, Malcolm Starkey, Randall J. Lee, Peter P. Rainer, Philip M. Hansbro, Andrew J. Boyle

**Affiliations:** 1 School of Medicine and Public Health, The University of Newcastle, Callaghan, NSW, Australia; 2 Hunter Medical Research Institute, New Lambton Heights, NSW, Australia; 3 School of Biomedical Sciences and Pharmacy, The University of Newcastle, Callaghan, NSW, Australia; 4 Priority Research Centre’s for Healthy Lungs and GrowUpWell, School of Biomedical Sciences and Pharmacy, The University of Newcastle, Callaghan, NSW, Australia; 5 Department of Cardiovascular Medicine, John Hunter Hospital, New Lambton Heights, NSW, Australia; 6 Department of Medicine, Division of Cardiology, University of California San Francisco, San Francisco, CA, United States of America; 7 Edyth and Eli Broad Center for Regenerative Medicine and Stem Cell Research, University of California San Francisco, San Francisco, CA, United States of America; 8 Division of Cardiology, Medical University of Graz, Graz, Austria; 9 Centre for inflammation, Centenary Institute, Sydney, NSW, Australia; 10 University of Technology, Faculty of Science, Ultimo, NSW, Australia; University of Central Florida, UNITED STATES

## Abstract

**Introduction:**

The prevalence of heart failure increases in the aging population and following myocardial infarction (MI), yet the extracellular matrix (ECM) remodeling underpinning the development of aging- and MI-associated cardiac fibrosis remains poorly understood. A link between inflammation and fibrosis in the heart has long been appreciated, but has mechanistically remained undefined. We investigated the expression of a novel protein, extracellular matrix protein 1 (ECM1) in the aging and infarcted heart.

**Methods:**

Young adult (3-month old) and aging (18-month old) C57BL/6 mice were assessed. Young mice were subjected to left anterior descending artery-ligation to induce MI, or transverse aortic constriction (TAC) surgery to induce pressure-overload cardiomyopathy. Left ventricle (LV) tissue was collected early and late post-MI/TAC. Bone marrow cells (BMCs) were isolated from young healthy mice, and subject to flow cytometry. Human cardiac fibroblast (CFb), myocyte, and coronary artery endothelial & smooth muscle cell lines were cultured; human CFbs were treated with recombinant ECM1. Primary mouse CFbs were cultured and treated with recombinant angiotensin-II or TGF-β1. Immunoblotting, qPCR and mRNA fluorescent in-situ hybridization (mRNA-FISH) were conducted on LV tissue and cells.

**Results:**

ECM1 expression was upregulated in the aging LV, and in the infarct zone of the LV early post-MI. No significant differences in ECM1 expression were found late post-MI or at any time-point post-TAC. ECM1 was not expressed in any resident cardiac cells, but ECM1 was highly expressed in BMCs, with high ECM1 expression in granulocytes. Flow cytometry of bone marrow revealed ECM1 expression in large granular leucocytes. mRNA-FISH revealed that ECM1 was indeed expressed by inflammatory cells in the infarct zone at day-3 post-MI. ECM1 stimulation of CFbs induced ERK1/2 and AKT activation and collagen-I expression, suggesting a pro-fibrotic role.

**Conclusions:**

ECM1 expression is increased in ageing and infarcted hearts but is not expressed by resident cardiac cells. Instead it is expressed by bone marrow-derived granulocytes. ECM1 is sufficient to induce cardiac fibroblast stimulation *in vitro*. Our findings suggest ECM1 is released from infiltrating inflammatory cells, which leads to cardiac fibroblast stimulation and fibrosis in aging and MI. ECM1 may be a novel intermediary between inflammation and fibrosis.

## Introduction

Heart failure (HF) causes significant morbidity and mortality, and aging is the strongest risk factor for HF due to its association with inflammation and progressive fibrosis [1–4]. Cardiac fibrosis is the result of the net accumulation of extracellular matrix (ECM) proteins during tissue remodelling, which causes myocardial stiffness and significantly impairs cardiac structure and function, and leads to HF [3, 5–12]. Fibrosis is a hallmark of the aging and infarcted heart, however the mechanisms underpinning the development of fibrosis are disease-specific [13]. Two of the most common cardiac heart failure models are pressure-overload induced cardiomyopathy and myocardial infarction (MI) [14–16]. Pressure-overload cardiomyopathy induces fibrosis as the result of large scale hemodynamic stress and left ventricle (LV) hypertrophy [17]. MI is associated with intense early inflammation, followed by ECM remodeling of the infarct zone and adjacent tissue, leading to collagen accumulation and fibrosis. This alters cardiac structure and function with an increased likelihood of heart failure [12, 18, 19]. The aggressive inflammation that occurs post-MI is well documented and referred to as the ‘inflammatory phase’ (~0–3 days post-MI). This is followed by a ‘proliferative phase’ (~2–7 days post-MI) which consists of fibrotic tissue deposition and angiogenesis, and a ‘maturation phase’ (~7+ days post-MI) which consists of ECM crosslinking and vascular maturation [20, 21].

Currently, the molecular events underpinning ECM remodeling are incompletely understood. As a result, there are few effective treatments for cardiac fibrosis. Extracellular matrix protein 1 (ECM1) is a widely expressed 85-kDa glycoprotein protein encoded by the ECM1 gene located at chromosome 1q21 in humans [22]. ECM1 is a multifunctional protein that interacts with the majority of other ECM proteins. Through these interactions, ECM1 enhances ECM protein binding and mediates a variety of processes in many tissues. The processes involved include the regulation of dermal-epidermal ECM integrity and barrier function [23–25], regulation of T helper lymphocyte type 2 (T_H_2) cell migration [26], inflammatory bowel disease [27], endochondral bone formation [28], stimulation of angiogenesis and tumour progression [29–32], and mediation of Trastuzumab resistance in breast cancer [33]. The cell types that express ECM1 are unknown, although it was initially identified to be expressed by the murine osteogenic stromal cell line MN7 [22], and later T_H_1 and T_H_2 lymphocytes [26]. Despite the abundant evidence highlighting its crucial role in other tissues, the role of ECM1 in cardiovascular disease is currently unknown.

Therefore, we studied ECM1 expression in aging and diseased hearts and explored the mechanisms of its actions in order to more clearly understand ECM remodeling and identify novel therapeutic targets to develop effective medical treatments for cardiac fibrosis.

## Materials & methods

### Mouse studies and ethics

Male C57Bl/6 mice were used for all experiments, with the exception of bone marrow cell (BMC) experiments where both male and female mice were used; young mice were 2–3 months old, aging mice were 18 months old. Mice were used with approval from the University of Newcastle’s animal care and ethics committee (ACEC, approval numbers A-2014-409 & A-2014-435), and according to the NIH guidelines and the guidelines of the Institutional Animal Care and Use Committee of UCSF the University of California. All experimental procedures conducted on mice were in strict accordance with the Australian Code of Practice for the Care and Use of Animals for Scientific Purposes. Animals were anesthetized with 5% isoflurane, and sacrificed vie 5% isoflurane anesthesia followed by exsanguination. All efforts were made to minimise animal suffering.

### Human studies and ethics

All patients provided written informed consent for tissue collection for experimental purposes, and all protocols were conducted in strict accordance with the Australian National Statement on Ethical Conduct in Human Research 2007, and approved by the University of Newcastle’s Human Research Ethics Committee (HREC); approval number H-2014-0390. Written consent was obtained, and right atrial appendage tissue was collected from ischemic heart disease patients undergoing coronary artery bypass grafting procedure.

### Inducing MI and pressure-overload in mice

MI was surgically induced by performing left anterior descending (LAD) ligation surgery [34], and pressure-overload by transverse aortic constriction (TAC) [17], as we have previously described in young mice. Briefly, mice were anesthetized, intubated and placed supine on a heat mat to maintain their temperature at 37°C. For induction of MI, a thoracotomy was performed, and the LAD ligated using a 7–0 prolene suture. For induction of pressure-overload cardiomyopathy, a 6–0 polypropylene suture was tied around a 27G needle placed over the aortic arch, and the needle was removed. For both LAD-ligation and TAC, the chest was then closed, and the animal allowed to recover to day-3 or day-28 post LAD-ligation and day-3 or week-13 post-TAC.

### Tissue preparation and analysis

Mice were humanely euthanized as previously described [35] and whole hearts were surgically removed and perfused with 0.5 mL saline through the aorta, and the LV was snap frozen and stored at -80°C. MI tissue was separated into three distinct zones prior to snap freezing depending on distance from the site of infarct: infarct zone, border zone, and remote zone. For TAC studies, the whole LV was used. mRNA and protein extraction and quantification methods are specified in S1 Text. Briefly, protein and mRNA was extracted, and subject to western blotting and quantitative polymerase chain reaction (qPCR) analysis respectively to analyze ECM1 expression, as we have previously described [36]; qPCR primer sequences listed in the S1 Table. All *ECM1* mRNA expression data is expressed as fold-change in delta delta threshold cycle (ΔΔCt) relative to control *ECM1* mRNA expression, and normalized to *Tpt-1* housekeeping gene. All western blot ECM1 protein expression data was normalized to either β-tubulin (55kDa) or β-actin (42kDa) as the loading control for relative band density analysis. An additional cohort of young healthy control mice were used for cardiac fibroblast (CFb) cell culture experiments. Protein was extracted from primary mouse and human CFbs and the following commercially sourced human cardiac cell lines: CFbs (Sigma Aldrich), coronary artery endothelial and smooth muscle cells (HCAEC & HCAESMC respectively; Sigma Aldrich), and human cardiac myocytes (HCM; PromoCell); n = 1 technical replicates/cell line. BMCs for the below mRNA fluorescent in-situ hybridization (mRNA-FISH) protocol were extracted as described by Liu and Quan [37]. BMCs were analyzed whole (n = 3), or separated in mononuclear and granulocyte cell fractions (n = 4) with Ficoll-Paque PREMIUM 1.084 (GE Healthcare) as per the manufacturer protocol. Frozen tissue sections (day-3 post-MI) to be used in mRNA-FISH were sectioned at 8μm using a Leica CM1950 cryostat at -20°C, as per the manufacturer protocol.

### Cell culture

Specific primary mouse and human CFb cell culture methods are outlined in S1 Text. Briefly, mouse primary CFbs were serum starved for 24 h, followed by treatment with either recombinant angiotensin-II (Ang-II; 100nM), transforming growth factor β1 (TGF-β1; 10ng/ml) or Complete CFb Media alone, for 48 h. Primary human CFb cells were cultured under standard conditions. Cell culture of commercially sourced human CFb, HCM, HCAEC and HCASMC cell lines were performed with recommended reagents as per the manufacturer’s protocols. Human CFbs (Sigma Aldrich) were serum starved for 24 h, then treated with recombinant ECM1 (20ng/ml) for 10, 30 or 50min to investigate ERK1/2, AKT and p38 pathway activation, or 48 hours to investigate collagen-I expression. Protein and mRNA were extracted from cells for use in SDS-PAGE, Western Blot and qPCR.

### mRNA-FISH

Oligonucleotide mRNA FISH probes were sourced from LGC Biosearch Technologies (Stellaris) for both ‘Ship Ready’ Mouse GAPDH, and custom Stellaris made Mouse *ECM1*; *ECM1* and GAPDH probe sets were conjugated with Quasar 670 dye. mRNA-FISH was conducted on fresh extracted BMCs, and cryostat-sectioned 8 μm frozen tissue sections as per the Stellaris ‘Cells in Suspension’ and ‘Frozen Tissue’ RNA FISH protocols. Cells and tissue were imaged using fluorescence microscopy (Zeis; Axio Imager M2) under DAPI and Cy5 channels to detect nuclear stain 4',6-Diamidino-2-Phenylindole, Dihydrochloride (DAPI) and Quasar 670 dye, respectively.

### Flow cytometry

Bone marrow cells were collected from 4 mice. Cells were blocked with Fc block (anti-CD16/32) for 30 min and stained with the antibodies depicted in Table 1. Staining and washing steps were performed with FBS stain buffer (554656, BD Biosciences). Samples were acquired on a BD LSR Fortessa X-20 flow cytometer.

**Table 1:** Flow cytometry antibody specifications.

Antibody	Catalog #	Clone	Fluorophore	Assay dilution	Source
Rat Anti-Mouse I-A/I-E (MHC2)	563414	M5/114.15.2	BV711	1/1000	BD Biosciences
Rat Anti-CD11b	562287	M1/70	PE-CF594	1/750	BD Biosciences
Rat Anti-Mouse F4/80	565635	T45-2342	BV480	1/100	BD Biosciences
Rat Anti-Mouse CD117	563399	2B8	BV650	1/200	BD Biosciences
Hamster Anti-Mouse CD11c	560584	HL3	PerCP-Cy5.5	1/100	BD Biosciences
Rat Anti-Mouse CD3	564010	17A2	BV786	1/50	BD Biosciences
Rat Anti-Mouse Ly-6C	562727	AL-21	BV421	1/200	BD Biosciences
Rat Anti-Mouse CD45	552848	30-F11	PE-Cy7	1/1000	BD Biosciences
Rat Anti-Mouse Ly-6G	561105	1A8	FITC	1/100	BD Biosciences
Hamster Anti-Mouse FcεRI-α	134308	MAR-1	PE	1/400	Biolegend

Flow cytometry data were analysed using FlowJo (version 10). Cells were gated using FSC-A vs SSC-A, and single-cells identified using FSC-A vs FSC-H. Of these populations, 50,000 cells from each animal were downsampled and concatenated to one .fcs file. t-distributed stochastic neighbour embedding (tSNE) analysis was performed on the concatenated sample containing the gated populations from all four samples combined. For this, all compensated channels were assessed (FITC, PerCP-Cy5.5, BV421, BV510, BV650, BV711, BV786, PE, PE-CF594, PE-Cy7, APC) under the following tSNE settings: Iteration 1000, Perplexity 20, Eta 200, Theta 0.5. Histograms established from the same plot show the differential expression of the cell markers in ECM1+ and ECM1- cells.

### Statistical analysis

Data are represented as mean ± standard deviation. Parametric data were analyzed using t-tests for two groups, or one-way ANOVA for more than two groups. Multiple comparisons were assessed for normalcy of distribution (parametric distribution) using Shapiro-Wilk tests. Non-parametric data were analyzed using Mann-Whitney tests, or Kruskal-Wallis tests for more than two groups. All tests were conducted with an assigned significance level of p<0.05.

## Results

### ECM1 is upregulated in aging and infarcted hearts, but not in pressure overload cardiomyopathy

**Fig 1:**
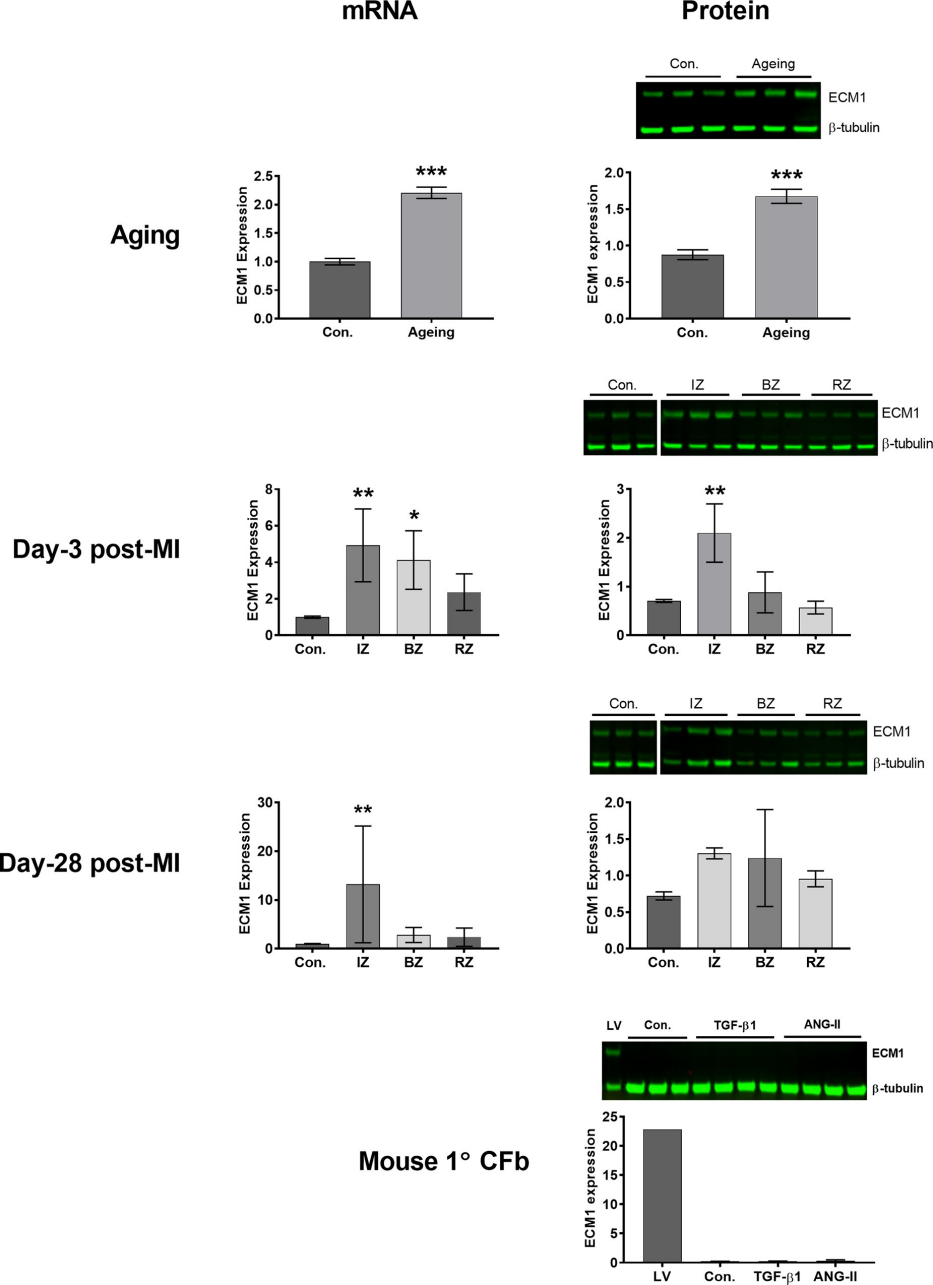
ECM1 mRNA and protein levels are upregulated in aging LV tissue and in the infarct zone day-3 post-MI, but ECM1 is not expressed by cardiac fibroblasts. ECM1 mRNA and protein (~75kDa) is upregulated in aging and specifically in the infarct zone of day-3 post-MI LV tissue. However, mouse primary cultured CFbs show no ECM1 expression under control conditions (Con.), nor in response to 48 h of treatment with recombinant TGF-β1 (10 ng/ml), or ANG-II (100nM) (left); positive control shows ECM1 expression in mouse LV (LV—left lane). Data is expressed as mean ± SD. Control: n = 3 mice for mRNA, post-MI: n≥6 mice/group for mRNA. Blot images of Day-3 post-MI, Day-28 post-MI and Mouse 1° CFb were cropped only to re-arrange the order of control lanes of the same blot. β-tubulin (~55kDa) is included as the loading control. All protein n = 3 mice/group for each pathological state, and n = 3 technical replicates/group for control Mouse 1° CFb, and n = 4 for TGF-β1 and ANG-II Mouse 1° CFb. *p<0.05; **p<0.01; ***p<0.001 relative to control.

*ECM1* mRNA (p = 0.0002) and protein (p = 0.0006) expression were significantly upregulated in aging compared to young LV (Fig 1). Following-MI, a significant upregulation of *ECM1* mRNA (p = 0.004) and protein (p = 0.005) occurred early in the ‘infarct zone’, relative to healthy non-infarcted LV. The ‘border zone’ showed upregulation in *ECM1* mRNA but not protein (mRNA, p = 0.02; protein, p = 0.89) and no significant difference was seen in ECM1 expression in the ‘remote zone’. Later, at day 28, these changes are beginning to resolve. There remained a slight numerical increase in ECM1 protein expression in the ‘infarct zone’, but this was no longer significant. Following TAC, however, no significant difference in ECM1 mRNA or protein expression was detected (S1 Fig). As ageing and MI are associated with inflammatory cell infiltration into the left ventricle, we hypothesized that inflammatory cells may be responsible for ECM1 expression.

### ECM1 is expressed in BMCs and infiltrating cells in the infarct zone post-MI

**Fig 2:**
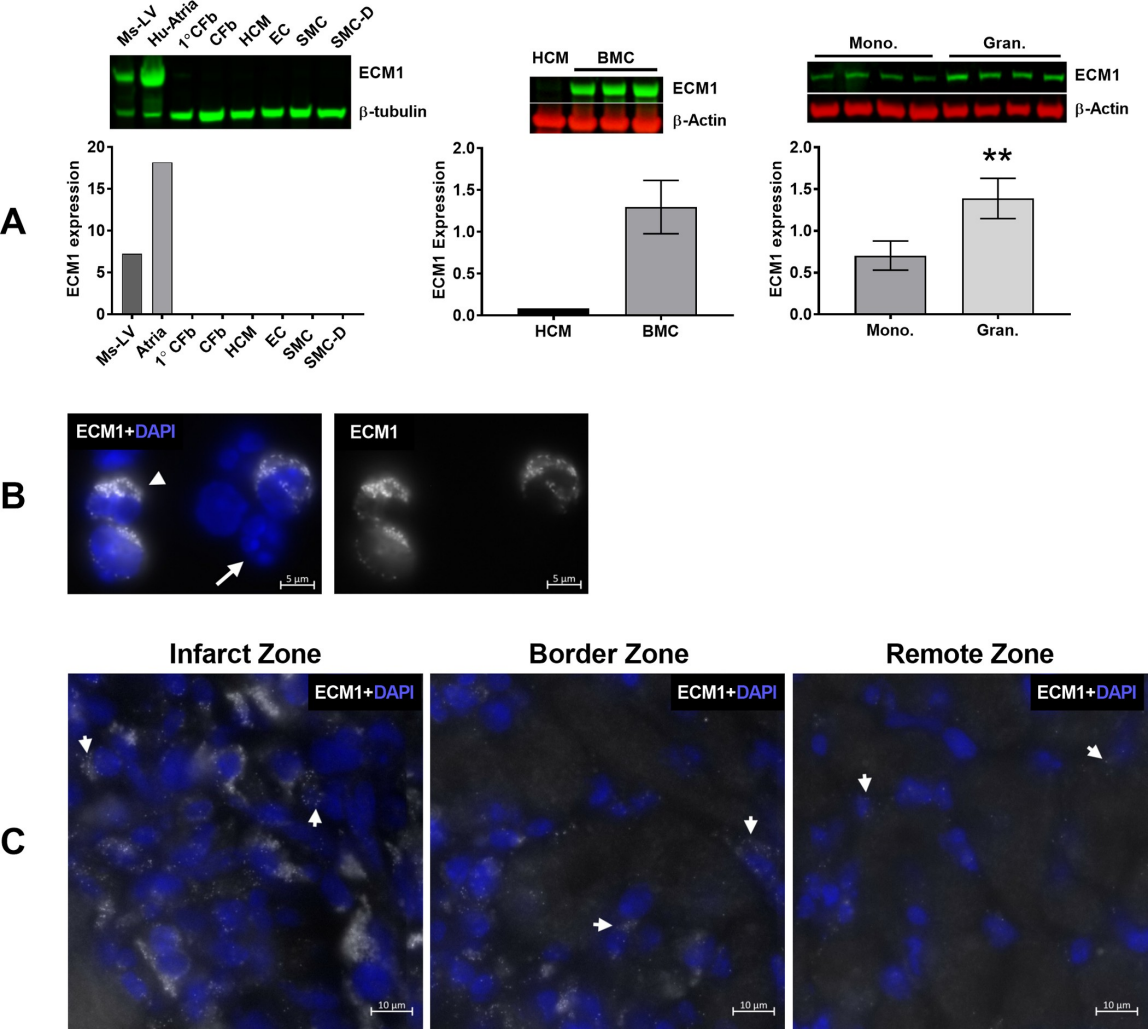
ECM1 is not expressed by resident cardiac cells, but is highly expressed in bone marrow cells (BMCs) and cells in the infarct zone day-3 post-MI. A) Immunoblot of ECM1 protein expression (~75kDa) in primary human CFbs, and cell lines: human CFb (CFb), human cardiac myocytes (HCM), human coronary artery EC (EC), SMC (SMC) and SMC differentiated (SMC-D) cell lines (Left). No ECM1 protein expression is seen in any cell type; positive controls show ECM1 expression in mouse LV (Ms-LV), and human atrial appendage (Hu-Atria), all data was normalized to β-tubulin expression as a loading control (~55kDa). Immunoblot of ECM1 protein expression in BMCs from young healthy mice (middle, n = 3, HCM serve as a negative control), and in BMCs separated into mononuclear and granulocyte fractions by Ficoll-Paque gradient density centrifugation (right, n = 4). A high level of ECM1 expression in BMCs, with a significantly higher level of ECM1 expression in granulocytes (gran.), compared to monocytes (mono., p = 0.005); all data was normalized to β-Actin expression as a loading control (~42kDa). B) mRNA-FISH of *Ecm1* mRNA (white), conducted on BMCs in suspension; DAPI nuclear stain was used to visualize cell nuclei (Blue). *ECM1* mRNA is expressed in some BMCs (arrowhead), and not in other BMCs (full-arrow). C) mRNA-FISH of *ECM1* mRNA conducted on day-3 post-MI LV tissue, showing positive *ECM1* expression as areas of sharp and punctate Cy5 fluorescent dots; example areas indicated by arrowheads, DAPI nuclear stain was used to visualize cell nuclei (Blue). High *ECM1* mRNA expression is seen in the infarct zone, some expression in the border zone, and little expression in the remote zone, consistent with inflammatory cell infiltrate. In Fig 2A, positive controls and resident cardiac cells (right) n = 1 technical replicates/group, BMCs n = 3/group, BMC monocytes and granulocytes n = 4/group. **p<0.01 relative to control.

The cellular origin of ECM1 observed in LV samples remained unknown. We identified that primary mouse CFbs do not express ECM1 protein under standard culture conditions, nor when stimulated with recombinant TGF-β1 (10ng/ml) or ANG-II (100nM) (Fig 1). We investigated if ECM1 originated from other resident cardiac cells, or infiltrating inflammatory cells in the heart. We cultured each of the known resident cell types in the heart separately to assess ECM1 expression: primary human CFbs (1°CFb), and human CFb cell line, human cardiac myocytes (HCM), human coronary artery endothelial cells (HCAEC), human coronary artery smooth muscle cells (HCASMC) and differentiated-HCASMC cell lines were cultured under standard conditions and analyzed for ECM1 protein expression. No ECM1 protein was detected from any cell type (Fig 2A). To investigate the possibility that ECM1 is of inflammatory cell origin in the heart, bone marrow cells (BMCs) were then extracted from young healthy mice. ECM1 protein was highly expressed in BMCs, and when separated into monocyte and granulocyte cell fractions, there was significantly more ECM1 expression in the granulocyte fraction of BMCs (Fig 2A). *ECM1* mRNA-FISH was then conducted on BMCs to confirm mRNA expression (Fig 2B), followed by mRNA-FISH on frozen LV tissue sections from day-3 post-MI animals to further investigate the cellular origin of ECM1 (Fig 2C). *ECM1* mRNA was highly localized to the dense inflammatory cell infiltrate of the infarct and border zones. Only minor expression of *ECM1* was seen surrounding myocytes of the remote zone, with no *ECM1* expression seen within cardiac myocytes.

**Fig 3:**
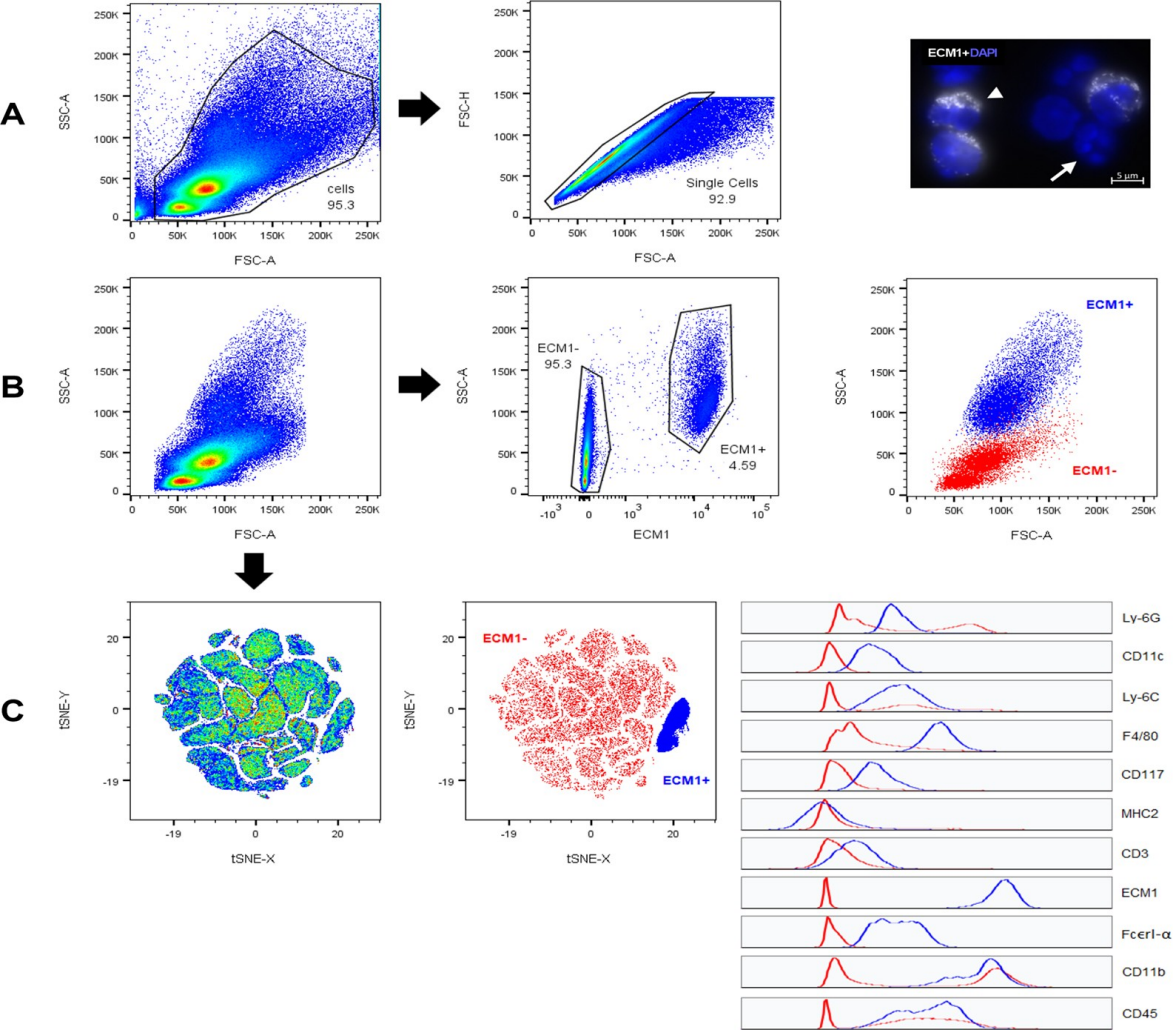
ECM1 FISH-Flow cytometry. ECM1 mRNA-FISH was coupled with flow cytometry to identify the cell surface marker profile of ECM1 positive (ECM1+) cells in young healthy mouse bone marrow. A) Forward scatter area (FSC-A; x-axis) versus side scatter area (SSC-A; y-axis) pseudocolour plot, demonstrating the gating strategy used to define our “cells” population, in order to exclude debris; left graph. Forward scatter area (FSC-A; x-axis) versus forward scatter height (FSC-H; y-axis) pseudocolour plot, demonstrating the gating strategy used to define “single cells” population, in order to exclude cell doublets; middle graph. An image of mRNA-FISH of *Ecm1* mRNA (white), conducted on BMCs in suspension; DAPI nuclear stain was used to visualize cell nuclei (Blue); right image. B) FSC-A versus SSC-A pseudocolour plot of the defined “single cells” population (left graph), and ECM1 (APC area channel) versus SSC-A pseudocolour plot demonstrating the gating strategy used to define ECM1 positive (ECM1+) and ECM1 negative (ECM1-) cells (middle graph). Included is FSC-A versus SSC-A of the ECM1+ gate overlayed onto the “single cells” population. C) A pseudocolour plot of cell population clusters after tSNE analysis conducted on the concatenated sample containing the gated populations from all four biological replicate samples combined (left graph; presented as tSNE x-axis versus tSNE y-axis). All compensated channels were assessed: FITC (Ly-6G), PerCP-Cy5.5 (CD11c), BV421 (Ly6C), BV510 (F4/80), BV650 (CD117), BV711 (MHC2), BV786 (CD3), APC (ECM1), PE (FcεRI-α), PE-CF594 (CD11b), and PE-Cy7 (CD45). Gates corresponding to ECM1+ (blue) and ECM- (red) cells from the concatenated sample was overlayed onto the tSNE pseudocolour graph (middle graph) to visualise expression levels of all cell surface markers investigated, as represented by the corresponding histograms (right graph); x-axis corresponds to expression level, y-axis corresponds to cells number.

To further investigate the cellular source of ECM1+ cells, multicolour FISH-flow cytometry was conducted on bone marrow cells, which had been tagged with an ECM1 mRNA-FISH probe, alongside fluorescently labelled antibodies specific to the following cell surface markers: Ly-6G, CD11c, Ly-6C, F4/80, CD117, MHC2, CD3, FcεrI-α, CD11b and CD45. Following tSNE analysis, ECM1+ cells demonstrated expression (to varying levels) for CD45, CD11b, CD11c, F4/80, Ly6-C, Ly-6G, and FcεrI-α, (Fig 3). Further, when visualising side scatter (SSC) versus forward scatter (FSC) for ECM1+ cells, it is clear that ECM1+ cells are highly granular and moderate to large in size, indicative of granulocytes (e.g. neutrophils, eosinophils) or large cells such macrophages. Taken together, these results suggest that ECM1 is constitutively expressed in granular bone marrow cells but not in smaller CD3+ or MHC II+ cells. Following MI, ECM1+ cells may be recruited to the infarct zone of the LV to contribute to post-inflammatory fibrosis.

### ECM1 stimulates collagen production from cardiac fibroblasts via activation of ERK1/2 and AKT

**Fig 4:**
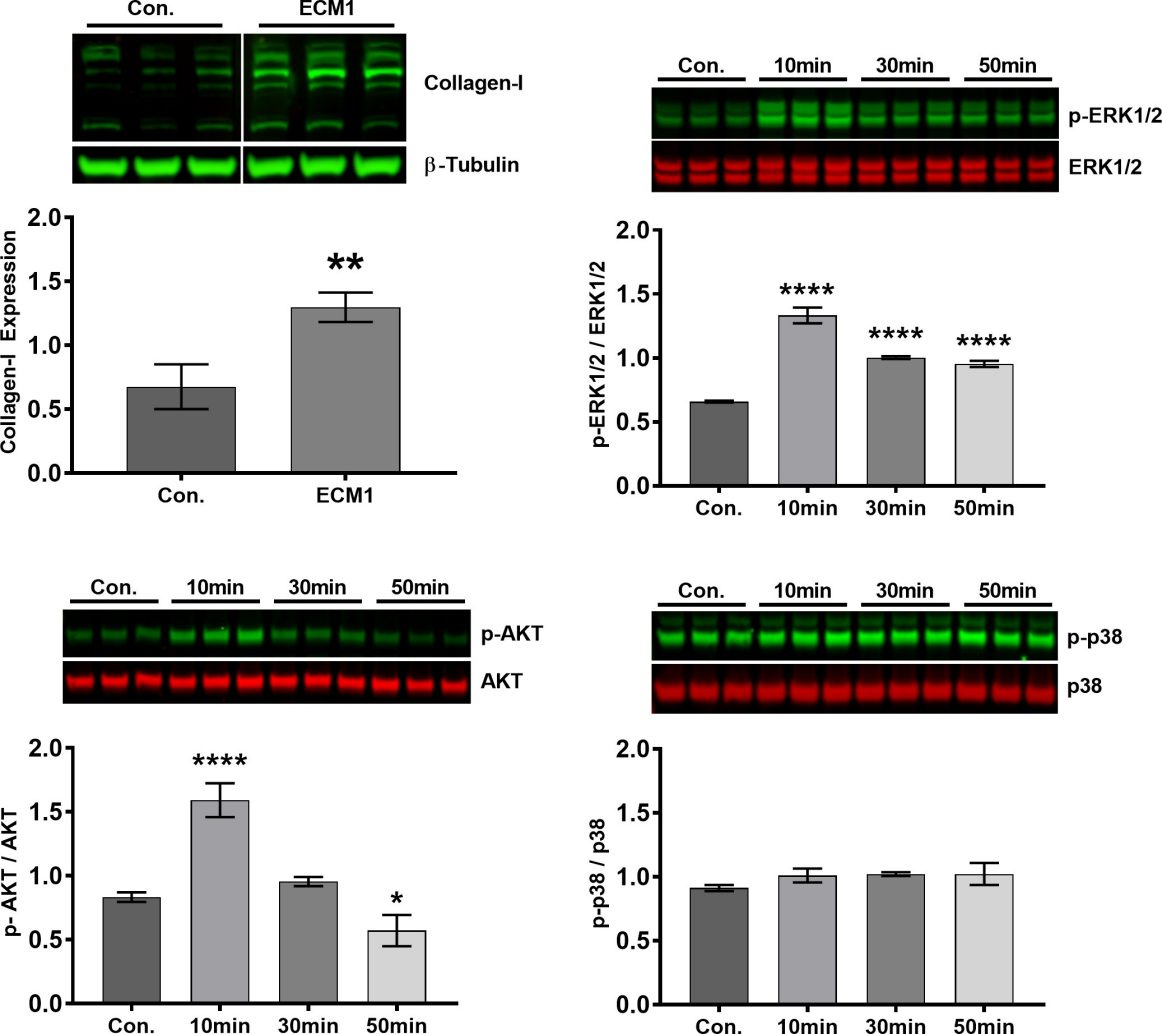
ECM1 stimulates collagen-I expression and ERK1/2 and AKT pathway activation in cardiac fibroblasts. Collagen-I protein expression was increased in human CFbs in response to 48 h of treatment with recombinant ECM1 (20ng/ml) relative to untreated control (Con.) cells (top left); data normalized to β-tubulin expression (~55kDa). Immunoblots of ERK1/2 and p-ERK1/2 at Thr202/Tyr204 (top right), AKT and p-AKT at Ser473 (bottom left), and p38 and p-p38 at Thr180/Tyr182 (bottom right) expression in human CFbs treated with recombinant ECM1 (20ng/ml) for 10, 30 or 50 minutes. Shows significant ERK1/2 activation at 10, 30 and 50min, and AKT activation at 10min. Data is represented as phosphorylated/non-phosphorylated protein. The blot image in top left figure was cropped only to re-arrange the order of control lanes of the same blot. All data is expressed as mean ± SD, with n = 3 technical replicates/group. *p<0.05, **p<0.01, ****P<0.0001 relative to control.

Based on our findings of excess fibrosis and increased ECM1 expression in the aging and post-MI LV, we explored whether ECM1 contributes to the observed fibrosis. Recombinant ECM1 was added to human CFb in culture and was sufficient to increase collagen production (p = 0.004, Fig 4). To investigate the mechanism of collagen-I expression, we assessed several key signalling pathways involved in fibrosis (AKT, ERK1/2 & p38). Recombinant ECM1 significantly upregulated human CFb ERK1/2 activation (Thr202/Tyr204; p<0.0001) and AKT activation (Ser473) at 10 minutes (p<0.0001), and significantly downregulated AKT activation at 50 minutes (p = 0.02) compared to untreated control human CFbs (Fig 4). No significant difference in p38 activation (Thr180/Tyr182) was seen at any time-point.

## Discussion

Here, we identified a novel association of ECM1 with cardiac aging and MI. ECM1 was upregulated in the aging LV, and specifically in the infarct zone during the inflammatory phase early post-MI. Interestingly, there was no significant difference in ECM1 protein later post-MI, suggesting a time-dependent expression profile of ECM1 post-MI. We showed that ECM1 was not expressed by any tissue resident cell type within the heart but was highly expressed in BMCs, with a greater extent of ECM1 expression in granulocytes compared to monocytes. *ECM1* mRNA was visualized almost exclusively in inflammatory cells in the infarct zone and infiltrating the border zone during the inflammatory phase day-3 post-MI. ECM1 is sufficient for CFbs to produce collagen-I, supporting the hypothesis that ECM1 expression in the heart originates from infiltrating inflammatory cells and contribute to cardiac fibrosis via activation of ERK1/2 and/or Akt. A link between inflammation and fibrosis has long been appreciated, but has mechanistically remained undefined. These findings propose a novel paradigm in which ECM1, released from infiltrating inflammatory cells recruited from the bone marrow, acts on fibroblasts resulting in collagen secretion.

Aging has been associated with cardiac fibrosis as the result of a multitude of factors including cellular senescence, cell death and inflammation due to age-associated immunoinflammatory dysregulation [12, 38–40]. Here we identified an increase in ECM1 expression in aging mouse LV tissue. This is the first detection of ECM1 expression in the aging heart. Previous studies have identified an inverse trend in the dermis where ECM1 expression decreases in aging human skin [41, 42]. This opposing finding is likely due to the fact that the aging of human skin is associated with decreased collagen and elastin content in the ECM leading to fragmentation and disorganization of the remaining collagen fibres [42, 43]. In contrast, the aging heart is associated with the accumulation of collagen, elastin and other ECM proteins [38, 40], and a general shift in the expression of various matrix metalloproteinases (MMPs) [44]. ECM1 has been identified to bind many ECM proteins, and is described as a multifunctional binding core, and a biological glue [25, 33, 42, 45]. Thus, it is likely that ECM1 plays a role in conferring structural stability to the cardiac ECM via its protein-protein binding capacity, particularly in age-associated fibrotic ECM remodelling; similar to the previously proposed role of ECM1 in the skin [42]. However, as ECM1 is expressed by T_H_1/2 lymphocytes, it is also possible that the increase in ECM1 expression is a result of inflammatory cell infiltration to the myocardium with aging [2, 35, 46].

We identified upregulation of ECM1 protein and mRNA at day-3 post-MI during the inflammatory phase, selectively in the infarct zone. Day 1–3 post-MI is associated with acute inflammation involving infiltration of lymphocytes, eosinophils, and neutrophils to the site of infarct [20, 47–49]. One previous study has identified ECM1 expression in the heart during chronic sustained hypoxia, with an associated increase in LV inflammation and *ECM1* mRNA expression [50]. This correlates with our results at day-3 post-MI and suggests that ECM1 plays a role in the post-MI inflammatory response. Although not focussed on cardiac disease, another study identified ECM1 to be highly and selectively expressed by the T_H_2 subset of lymphocytes, and implicated ECM1 in controlling their egress from lymph nodes to the site of inflammation in the respiratory system; ECM1 deletion resulted in significantly reduced allergic airway inflammation *in vivo*, with reduced infiltration of eosinophils, macrophages and lymphocytes [26]. Of particular interest is that ECM1 expression in T_H_2 lymphocytes occurred only at day-3 post-antigen recognition and initiation of an immune response, the same time-point of our identified ECM1 upregulation post-MI. This correlates with our findings and suggests that ECM1 may play a similar role in the acute inflammatory response within 3 days post-MI: facilitating the migration of T_H_2 lymphocytes, eosinophils and macrophages to the site of infarct to promote the development of a fibrotic wound. This is plausible as T_H_2 lymphocytes (and other T lymphocytes) are crucial in promoting cardiac fibrosis through the expression of growth factors, chemokines & cytokines and/or by direct cell-cell interaction with fibroblasts to stimulate their activation, proliferation, trans-differentiation and matrix generation [13, 51–54]. Our data suggests that the large granular cells from the bone marrow, rather than lymphocytes, are responsible for ECM1 expression.

At 4–7 days post-MI during the transition into the proliferative phase, the inflammatory response is halted and inflammatory cells are cleared from the infarct zone [20, 55]. The maturation phase that follows at day-28 post-MI is associated with collagen crosslinking, ECM stabilization, and fibroblast cell clearance from the site of infarct [20, 21, 55]. Herein we saw the increase in ECM1 expression was largely attenuated by day-28. Our findings suggest an early inflammatory role of ECM1, rather than a structural role in post-MI fibrotic wound healing.

We showed that ECM1 is not detected in resident cardiac cells, but is highly expressed in BMCs, with a significantly higher level of ECM1 expression in granulocytes compared to monocytes. Further, multicolour FISH-Flow cytometry revealed ECM1+ bone marrow cells are granular, and of medium to large size. We then identified for the first time that ECM1+ cells are: Ly-6G-intermediate, CD11c-low, Ly-6C-intermediate, F4/80+, CD117-/low, MHC2-, CD3-, FcεrI-α+, CD11b+ and CD45+. Based on our findings, it appears that ECM1+ cells in the bone marrow of healthy, young mice are granulocytes or macrophages (e.g. neutrophils, eosinophils, mast cells) and likely not lymphocytes. No previous studies have been conducted on ECM1+ cells in bone marrow of young healthy animals, however literature has identified significant ECM1 expression in T_H_2 lymphocytes (at day-3 post antigen presentation). Although our findings are indicative of granulocytes, cells residing in the bone marrow may have different expression of both ECM1, as well as cell surface markers, compared to cells which have migrated to the infarct zone of the heart. Future studies will need to address the identity of ECM1+ cells in the infarct zone post-MI, and should include functional cell morphology data to provide a clear insight into the cellular origin of ECM1 in the heart.

Collectively, our results suggest that ECM1 expression in the heart is of inflammatory cell origin, whether they be cardiac-resident inflammatory cells and/or infiltrating inflammatory cells, and identifies a novel mechanism of multicellular signalling crosstalk via ECM1. This is plausible as migration and phenotype shift of BMCs and other inflammatory cells to the site of infarct during the inflammatory phase early post-MI is well established [56–59]. Further, inflammatory cells including lymphocytes, monocytes, neutrophils, macrophages and eosinophils, have been identified to play pivotal roles in orchestrating ECM remodelling and fibrosis in ischaemic wound healing in the infarct zone [47–49, 59]. Our *in vitro* findings correlate with our observations of *in vivo* upregulation of ECM1 at day-3 post-MI during the inflammatory phase, and considering other studies, suggest that ECM1 upregulation in the infarct zone day-3 post-MI is due to infiltrating inflammatory cells.

We then demonstrated that recombinant ECM1 elicits a rapid activation of ERK1/2 and AKT in CFbs, resulting in a downstream increase in collagen-I production. This is the first report of ECM1 dependent CFb cell signalling, and demonstrates that ECM1 stimulates cardiac fibroblast proliferative and pro-fibrotic pathways (AKT and ERK1/2, respectively), which may contribute to fibrotic tissue formation with aging, and post-MI *in vivo* [60–64]. Future studies are needed to elucidate: the specific inflammatory cell(s) of origin of ECM1, the mechanism of migration of these cells to the myocardium, the mechanism of stimulating ECM1 expression, as well as the mechanism of ECM1 to CFb interaction and cell signalling to stimulate this novel fibrotic response.

## Conclusion

Collectively, our findings suggest that ECM1 is expressed by infiltrating inflammatory cells, not resident cardiac cells, and is sufficient for fibroblast stimulation and collagen production *in vitro*. This suggests that ECM1 plays a role in cardiac fibrosis in aging and myocardial infarction. ECM1 may be a novel intermediary between inflammation and fibrosis and a novel therapeutic target.

## Supporting information

S1 Text Supplementary methods. (DOCX)

S1 Table qPCR target and reference gen primer specifications. (DOCX)

S1 Fig ECM1 expression Post-TAC. ECM1 mRNA and protein (~75kDa) is not differentially expressed at day-3 post-TAC, nor at week-13 post-TAC when compared to control. Data is expressed as mean ± SD. Control: n = 3 mice for mRNA and protein, post-TAC: n = 3 mice/group for protein, n = 4 mice/group for mRNA. Blot image was cropped only to re-arrange the order of control lanes of the same blot. β-tubulin (~55kDa) is included as the loading control.
(TIF)

S2 Fig Entire uncropped western blot images. (TIF)
