# Supplementary material for: Novel role of extracellular matrix protein 1 (ECM1) in cardiac aging and myocardial infarction
Source: PLoS One. 2019 Feb 21;14(2):e0212230. doi: 10.1371/journal.pone.0212230 (PMC6383988; doi:10.1371/journal.pone.0212230)
Supplement: S1 Text — (DOCX) [file pone.0212230.s001.docx]

**S1 Text. Supplementary methods.**

Novel Role of Extracellular Matrix Protein 1 (ECM1) in Cardiac Aging- and Myocardial Infarction

Sean A. Hardy^a,d^, Nishani S. Mabotuwana^a,d^, Lucy A. Murtha^a,d^, Brianna Coulter^a,d^, Sonia Sanchez-Bezanilla^b,d^, Mohammed S. Al-Omary^a,d,e^, Tharindu Senanayake^a^, Malcolm Starkey^b,c,d^, Randall J. Lee^e,f^, Peter P. Rainer^h^, Philip M. Hansbro^b,c.d^, Andrew J. Boyle^a,d,e^

1. School of Medicine and Public Health, The University of Newcastle, Callaghan, NSW, Australia
2. School of Biomedical Sciences and Pharmacy, The University of Newcastle, Callaghan, NSW, Australia
3. Priority Research Centre’s for Healthy Lungs and GrowUpWell, School of Biomedical Sciences and Pharmacy, The University of Newcastle, Callaghan, NSW, Australia
4. Hunter Medical Research Institute, New Lambton Heights, NSW, Australia
5. Department of Cardiovascular Medicine, John Hunter Hospital, New Lambton Heights, NSW, Australia
6. Department of Medicine, Division of Cardiology, University of California San Francisco, San Francisco, CA, USA
7. Edyth and Eli Broad Center for Regenerative Medicine and Stem Cell Research, University of California San Francisco, San Francisco, CA, USA
8. Division of Cardiology, Medical University of Graz, Graz, Austria

**Corresponding author:**

**Corresponding author:** Andrew J. Boyle, MBBS, PhD, FRACP, FACC, FAHA. Department of Cardiovascular Medicine, John Hunter Hospital. Locked Bag 1, HRMC Newcastle, NSW 2310, Australia. Email: [Andrew.boyle@newcastle.edu.au](mailto:Andrew.boyle@newcastle.edu.au) - Phone: +61249214205

**Short title:** ECM1: A novel mediator of cardiac fibrosis

**Declarations of interest: none**

**S1 Text. Supplementary Methods**

- 1. **Protein extraction and quantification**

To isolate total protein for use in SDS-PAGE, samples were suspended in RIPA lysis buffer containing Roche cOmplete™, Mini, EDTA-free protease inhibitor and PhosSTOP™ phosphatase inhibitor cocktail (Sigma-Aldrich®), and homogenized using a Precellys®24 high-throughput tissue homogenizer as per the manufacturer protocol. Concentration of protein lysate was quantified via BCA assay using a Pierce™ BCA Protein Assay Kit (ThermoFisher Scientific) as per the manufacturer protocol.

- 1. **RNA isolation and analysis**

RNA isolation was conducted using TRIzol® Plus RNA purification reagents (Life Technologies) as per the manufacturer protocol. RNA samples were subject to DNase I treatment using a DNase I, Amplification Grade Kit (ThermoFisher scientific) as per the manufacturer protocol. The NCBI Primer-BLAST program was used to identify exon-spanning primers specific to each gene of interest in order to avoid any amplification of genomic DNA. Primers which satisfied the set parameters were screened using ‘Primer3’ software version 2.3.7 (Koressaar and Remm, 2007; Untergasser et al., 2012). All forward and reverse primer sequences, amplicon length, and accession numbers are outlined in Table 2.1. Reverse transcription PCR (RT-PCR) reactions for complementary DNA (cDNA) synthesis were conducted using a GeneAmp® PCR System 9700 (Applied Biosystems). Reverse transcription (RT) and cDNA synthesis was conducted with 200ng of RNA each from each individual sample using Oligo (dT)18 primers (Bioline), dNTP (Bioline) and a SuperScript® III (Invitrogen) First Strand Synthesis System for RT-PCR.

All target and reference genes from cDNA transcripts were measured using quantitative real-time polymerase chain reaction (qPCR), with SYBR™ Green Master Mix (Life Technologies) reagents; amplification reactions were run using an Applied Biosystems® 7500 Real-Time PCR System (Applied Biosystems) instrument; 7500 Software v2.3 (Applied Biosystems). mRNA levels were quantified using the 2(-ΔΔct) method, normalized to Tpt-1 (for mouse mRNA) and β-actin (for human mRNA).

- 1. **SDS-PAGE and Western Blot**

SDS-PAGE was performed under reducing conditions. SDS sample buffer (MiliQ H_2_O supplemented with 8% SDS, 30% Glycerol, 0.02% Bromophenol Blue, 0.25m Tris-HCl; pH8) and Bolt™ sample reducing agent (Life Technologies) was added to total protein lysate solution. Samples were then reduced at 90 °C for 10 min, and 30 µL total volume loaded into each well of Precast Bolt™ 4-12% Bis-Tris Plus Gels (ThermoFisher Scientific). Gels were then placed in a Mini Gel Tank (Life Technologies) electrophoresis system containing Bolt™ MES SDS Running Buffer (Life Technologies), and 150V (constant volt) was applied for approximately 1h at room temp, or until the blue sample buffer had sufficiently run out of the gel casing. Gels were then transferred to nitrocellulose membranes. Membranes were blocked with Casein blocking buffer (Li-Cor Biosciences) for 1 hour at RT and incubated for 2 hours at RT or overnight at 4°C with either: ECM1 (1:300; SantaCruz® biotechnology H-300), Erk1/2 and p-Erk1/2 (1:2000 and 1:1000; Cell Signaling Tech., 4696S and 4370S), P38-MAPK and p-P38-MAPK (1:2000 and 1:500; Cell Signaling Tech., 9212S and 9216S), AKT and p-AKT MAPK (1:2000 and 1:500 Cell Signaling Tech., 40D4 and S473), collagen-I (1:250; Abcam ab21286) and loading controls β-tubulin (1:1000; Abcam ab2046), or β-Actin (1:20,000; Sigma-Aldrich® A3854) primary antibodies in Casein Blocking buffer. Membranes were washed in PBS supplemented with 0.01% tween-20 (w/v) (PBS-T) then incubated with IRDye 800CW Donkey anti-rabbit (1:15,000) and IRDye 680LT Donkey anti-mouse (1:20,000, Li-Cor Biosciences 926-68022) secondary antibodies in Casein blocking buffer for 1 hour at RT. Membranes were washed PBS-T and PBS, and imaged using a Li-Cor Odyssey CLx imaging system. Immunoblots were analysed using ImageJ software (version 1.49) to return a numerical value based on band density/intensity, to be used for quantification of protein expression as previously described (Abràmoff et al., 2004; Schneider et al., 2012).

All washing and incubation steps were performed on agitation. All casein blocking buffer used for dilution of antibodies was supplemented with 0.01% Tween-20 (w/v).

- 1. **Mouse primary cardiac fibroblast cell culture**

LVs were minced in 1x Digestion Media (200U/mL Collagenase II, 400µg/mL Trypsin in HBSS) and incubated at 37°C for 5 min. The first digestion supernatant was discarded, and the following 8 digestions were collected, filtered (100µm), diluted 1:2 in Complete Fibroblast Media (1x High Glucose DMEM supplemented with 10% foetal bovine serum, 2mM L-glutamine, 1mM sodium pyruvate, 100U/mL Penicillin, 100µg/mL Streptomycin), and centrifuged (300g, 10 min, 4°C). The pellet was resuspended in fresh media and plated for 2 hours to allow fibroblast adherence. Fresh media was applied and cultures maintained in a humidified incubator at 37°C with 5% CO_2_.

Cardiac fibroblast cells were cultured to passage 3, and stored at -80 °C in fibroblast storage media. For this project, cells were revived from passage 3, placed immediately into Complete Fibroblast Media, and cell suspension was transferred to Cellstar® 6-well cell culture plates (Greiner bio-one) at 100,000 live cells/well, and incubated at 37 °C with 5% CO_2_. Media was changed every 2 days until cells reached 80% confluence. Cells were passaged once, allowed to reach 80% confluence, then incubated with serum-free media for 24 h at 37 °C with 5% CO_2_, followed by treatment with either recombinant angiotensin-II (Ang-II; 100nM), transforming growth factor β1 (TGF-β1; 10ng/ml) diluted accordingly in complete fibroblast media, for 48 h.

To estimate live cell numbers, all cells were counted with a Countess™ Automated Cell Counter (Invitrogen™) as per the manufacturer protocol.

- 1. **Human primary cardiac fibroblast cell culture**

Explant primary culture was conducted on right atrial appendage tissue. Briefly, tissue was minced and incubated with agitation in 1X Digestion Media (200U/mL Collagenase II (Cat# 17101-015, ThermoFisher Scientific) and 400mg/mL Trypsin (Cat# 27250018, ThermoFisher Scientific) in HBSS (Cat# 14025092, Life Technologies) at 37°C for 30 min. 1X Digestion Media was quenched with Complete Fibroblast Media (DMEM/F12, Cat#11320033, Life Technologies) media supplemented with 20% foetal bovine serum (Cat#SFBS-F, Bovogen Biologicals), 1mM sodium pyruvate (Cat# 11360070, Life Technologies), and Pen/Strep (100U/mL Penicillin, 100mg/mL Streptomycin, Cat# 15070063, Life Technologies). Tissue was plated, and incubated in fresh Complete Fibroblast Media to allow cardiac fibroblasts to exit. Cultures were maintained in a humidified incubator at 37°C with 5% CO2. Cells were further expanded and protein extracted.

**References**

Abràmoff, M.D., P.J. Magalhães, and S.J. Ram. 2004. Image processing with ImageJ. *Biophotonics international*. 11:36-42.

Koressaar, T., and M. Remm. 2007. Enhancements and modifications of primer design program Primer3. *Bioinformatics (Oxford, England)*. 23:1289-1291.

Schneider, C.A., W.S. Rasband, and K.W. Eliceiri. 2012. NIH Image to ImageJ: 25 years of image analysis. *Nature methods*. 9:671-675.

Untergasser, A., I. Cutcutache, T. Koressaar, J. Ye, B.C. Faircloth, M. Remm, and S.G. Rozen. 2012. Primer3--new capabilities and interfaces. *Nucleic acids research*. 40:e115.
