# Supplementary material for: Novel role of extracellular matrix protein 1 (ECM1) in cardiac aging and myocardial infarction
Source: PLoS One. 2019 Feb 21;14(2):e0212230. doi: 10.1371/journal.pone.0212230 (PMC6383988; doi:10.1371/journal.pone.0212230)
Supplement: S1 Table — (DOCX) [file pone.0212230.s002.docx]

| **Gene** | **NCBI Gene Sequence Reference** | **Forward Primer** | **Reverse Primer** | **Amplicon Size (bp)** |
| --- | --- | --- | --- | --- |
| *Mouse ECM1* | NM_007899.2 | TAGTCCTGCCCGTGATGAGT | CCCTTCCACTTCCACAGAGC | 128 |
| *Mouse Tpt-1* | NM_009429.3 | ATGACGAGCTGTTCTCCGAC | AACACCGGTGACTACTGTGC | 182 |
| *Human ECM1* | NM_004425.3 | CTGCTGTGACCTGCCATTTC | TCCCCAGGACTCAGGTAACA | 135 |
| *Human β-Actin* | NM_001101.4 | AGAGCTACGAGCTGCCTGAC | AGCACTGTGTTGGCGTACAG | 184 |

**Supplementary Table 1: qPCR target and reference gene primer specifications.**
