# Supplementary figures and images for: Novel role of extracellular matrix protein 1 (ECM1) in cardiac aging and myocardial infarction
Source: PLoS One. 2019 Feb 21;14(2):e0212230. doi: 10.1371/journal.pone.0212230 (PMC6383988; doi:10.1371/journal.pone.0212230)

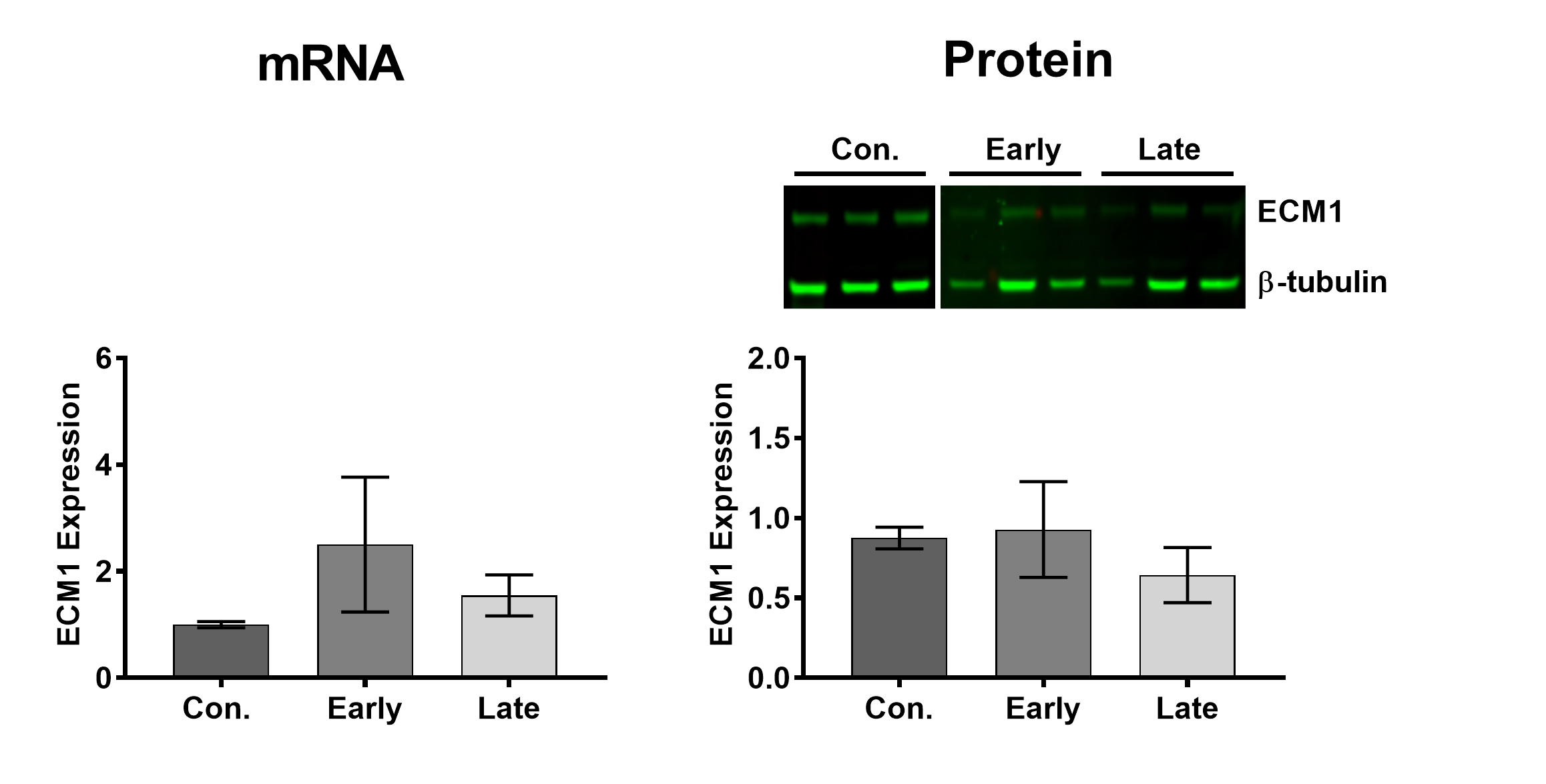

Supplement: S1 Fig — ECM1 mRNA and protein (~75kDa) is not differentially expressed at day-3 post-TAC, nor at week-13 post-TAC when compared to control. Data is expressed as mean ± SD. Control: n = 3 mice for mRNA and protein, post-TAC: n = 3 mice/group for protein, n = 4 mice/group for mRNA. Blot image was cropped only to re-arrange the order of control lanes of the same blot. β-tubulin (~55kDa) is included as the loading control. (TIF) [file pone.0212230.s003.tif]

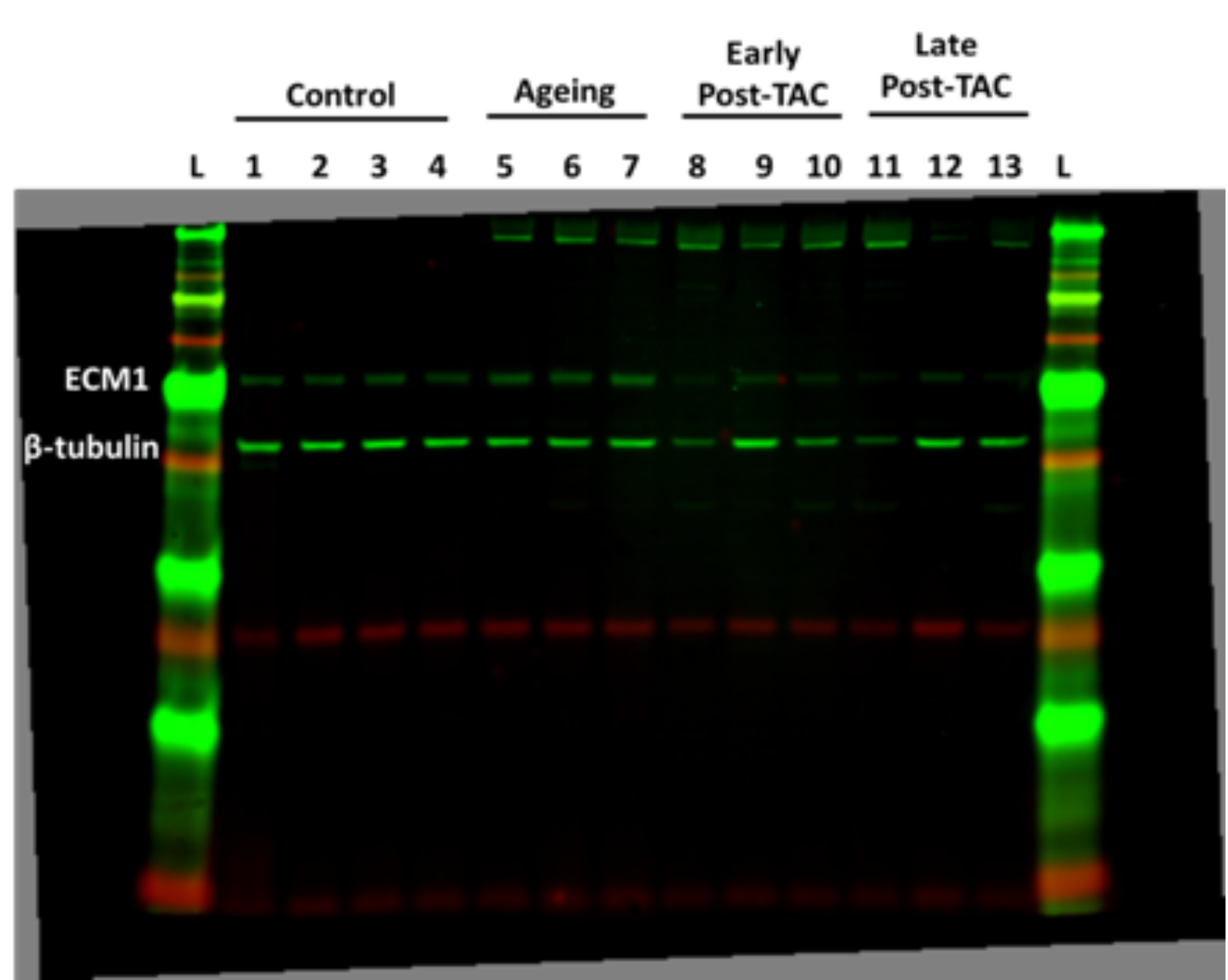

Supplement: S2 Fig — (TIF) [file pone.0212230.s004.tif]
